# A tailed mirtron promotes longevity in Drosophila

**DOI:** 10.1093/nar/gkad1158

**Published:** 2023-12-04

**Authors:** Sweta Khanal, Matthew de Cruz, Britton Strickland, Kody Mansfield, Eric C Lai, Alex Flynt

**Affiliations:** Cellular and Molecular Biology, School of Biological, Environmental, and Earth Sciences University of Southern Mississippi, USA; Cellular and Molecular Biology, School of Biological, Environmental, and Earth Sciences University of Southern Mississippi, USA; Cellular and Molecular Biology, School of Biological, Environmental, and Earth Sciences University of Southern Mississippi, USA; Cellular and Molecular Biology, School of Biological, Environmental, and Earth Sciences University of Southern Mississippi, USA; Department of Developmental Biology, Sloan Kettering Institute, USA; Cellular and Molecular Biology, School of Biological, Environmental, and Earth Sciences University of Southern Mississippi, USA

## Abstract

Thousands of atypical microRNAs (miRNAs) have been described in the genomes of animals; however, it is unclear if many of these non-canonical miRNAs can measurably influence phenotypes. Mirtrons are the largest class of non-canonical miRNAs that are produced from hairpins excised by splicing, which after debranching become substrates for Dicer and load into RISC. Most mirtrons require additional processing after splicing to remove ‘tail’ residues interposed between one of the host intron splice sites and base of the hairpin precursor structure. Despite most mirtrons requiring tail removal no function has been elucidated for a tailed species, indeed for all mirtrons identified function has only been assigned to a single species. Here we study miR-1017, a mirtron with a 3′ tail, which is well expressed and conserved in *Drosophila* species. We found that miR-1017 can extend lifespan when ectopically expressed in the neurons, which seems partly due to this miRNA targeting its host transcript, acetylcholine receptor Dα2. Unexpectedly we found that not only did miR-1017 function in *trans* but also in *cis* by affecting splicing of Dα2. This suggests a mechanism for mirtron evolution where initial roles of structural elements in splicing lead to secondary acquisition of *trans*-regulatory function.

## Graphical Abstract

**Graphical Abstract:**
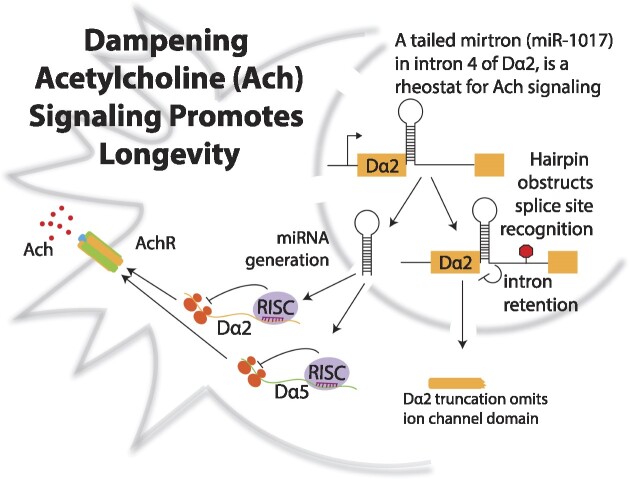

## Introduction

MicroRNAs are ∼22 nucleotide (nt) regulatory RNAs that post-transcriptionally modulate gene expression in eukaryotes through triggering transcript degradation or translational inhibition (1,2). miRNAs, comprising 1–5% of animal genes, regulate one-third of total transcripts and have been involved in a variety of biological pathways such as proliferation, differentiation, and metabolism (3). miRNAs are derived from hairpin structures often through cleavage by RNase III enzymes Drosha and Dicer (4). However, there are many non-canonical species that are generated via alternate biogenesis pathways, such as mirtrons which are derived from short hairpin introns (5–7). Mirtrons are Drosha independent, relying on the spliceosome for excision, and after debranching fold into a hairpin RNAs (8). Some mirtron hairpins span entirely their short host introns with splice sites defining the termini of both hairpin arms. These mirtrons will be referred to here as conventional mirtrons (9). Mirtrons can also be tailed where additional nt's separate hairpin structures from either the 5′ or 3′ splice site. Thus, only one terminus of the hairpin is defined by splicing and tail ‘trimming’ is needed to become a suitable dicer substrate. In *Drosophila*, the RNA exosome removes the 3′ tail of mir-1017 (10).

In some species, such as humans, tailed mirtrons outnumber conventional varieties however a role in gene expression has yet to be reported for any of these species (11). Further, mirtrons as a class have several fold lower expressions relative to canonical miRNAs and are poorly conserved. However, several studies have shown that conventional mirtrons can repress luciferase and fluorescence reporter in *Drosophila* and mammalian cells (8,12). In another study, artificial 3′-tailed mirtrons was able to reduce target expression in HEK293 cells (13). In loss of function studies, a single phenotype was found for a convention mirtron, miR-1010, which regulates its host transcript in a negative feedback loop (14). Together this suggests although mirtrons may be minor players in the overall collection of miRNAs, they have potential to influence gene expression and phenotypes. Nevertheless, several outstanding questions remain around functionality of tailed species and pressures that influence the distinct evolution of mirtrons relative to canonical miRNAs.

Here we investigate miR-1017, a well-expressed 3′-tailed mirtron conserved across *Drosophilids*. The host intron is located within the nicotinic acetylcholine receptor alpha-2 subunit, (nAchRa2/ Dα2) (Supplementary Figure 1). miR-1017 has also been assigned the dme-mirtron-1108 identifier (15). Interestingly this arrangement is similar to miR-1010 as miR-1017 is predicted to regulate Dα2 through a site in the open reading frame (16). To investigate the function of miR-1017, we first overexpressed this mirtron in neural tissue and found extended lifespan. An opposite phenotype was seen in publicly available miR-1017 deletion flies. Decrease in the mutant's lifespan could be rescued by an AchR antagonist suggesting perturbation of Ach signaling was indeed fundamental to this mirtron's phenotype (17). Unexpectedly, this deletion also abrogated splicing, leading to much greater perturbation of the host gene through truncation of the protein. Homozygotes for this deletion are viable indicating that full length Dα2 is not essential to *Drosophila* development, consistent with other reports (18). To isolate the phenotype of miR-1017, alleles were generated by genome editing. We observed similar decrease in lifespan yet very modest intron retention remained despite edits being only 2–4 nt changes. This prompted dissection of splicing regulation, which found the intron to be poorly defined and that many changes in the sequence could led to increased retention. Thus, the miR-1017 hairpin is not only able to produce *trans*-regulators, but it also participates in the logic of host intron splicing which may explain constraints on evolution and promote conservation of the mirtron.

**Figure 1.**
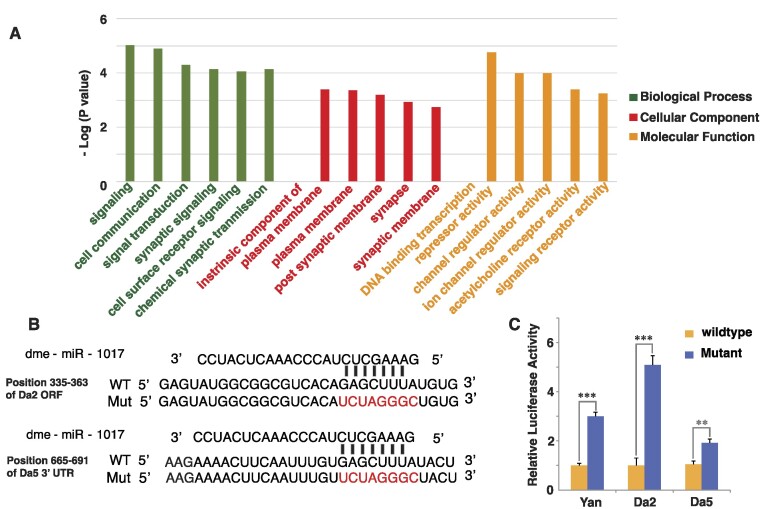
Validation of Dα2 and Dα5 transcripts as targets for miR-1017. (**A**) GO terms generated for the miR-1017 targets using GO Term finder showing higher cluster frequency as compared to genome frequency (*** indicates *P*-value < 0.01). (**B**) Generation of Renilla luciferase constructs bearing wildtype or mutant miR-1017 binding sites, for transcripts Dα2 and Dα5. Transversional mutations were used to generate the mutant binding site constructs. (**C**) Luciferase activity reveals mutation of miR-1017 binding sites results in elevated luciferase activity compared to wildtype constructs. Known target Yan and newly identified targets Dα2 and Dα5 present derepression 3-fold, 5-fold and 2-fold respectively.

## Materials and methods

### Fly acquisition and mutant flyline generation

Fly strains w^1118^, Elav-GeneswitchGAL4 (43642), UAS-ArcΑβ42 (33773), UAS-LUC-mir-1017/TM3 (41208) and mir-1017^KO^/TM3 (ID#58889) were obtained from the Bloomington stock center. miR-1017 deletion (miR-1017Δ) were generated as part of efforts to delete each miRNA from the *Drosophila* genome (19). Generation of miR-1017 mutants through gene editing used synthetic guide RNAs from Synthego and injection services from BestGene, 2 guide RNA targeting mature miR-1017 sequence were injected in embryos of nos-Cas9 flies (Supplementary Figure S4A). Upon eclosure of injected flies, they were crossed with balancer flyline (MKRS/TM6B) and F1 progenies were screened for mutation within miR-1017 sequence by PCR and sanger sequencing. Three fly lines (Mut1, Mut2 and Mut3) with SNPs or deletion within the miR-1017 mature miRNA sequence were found that lead to the disruption of the hairpin structure of the precursor. To generate transheterozygous lines, the mutant lines Mut1 and Mut2 were crossed to yield Mut1-2, Mut1 and Mut3 for Mut1-3, and Mut2 and Mut3 to generate Mut2-3.

### Cloning, transfection and RT-PCR

Mutant versions of miR-1017 were based on the previously published pUASt-DsRed-miR-1017. Mutants were generated through SOEing PCR-based mutagenesis and cloned using the cold fusion system (Systems Biosciences). miR-1017 sensors were generated by inserting synthetic fragments or RT-PCR amplified products into the Psicheck (Promega) vector by ligation. For luciferase assays S2 (R+) cells were co-transfected with either wildtype or mutant *renilla* constructs, pUASt-DsRed-mir-1017, and Ubi-GAL4 constructs using the TransIT-Insect reagent (Mirus). S2 *Drosophila* cells were plated 1 h before transfection and analyzed for luciferase activity 72 h after transfection with the dual luciferase assay (Promega) on a Synergy H1 microplate reader (BioTek). For splicing analysis, total RNA was extracted using Trizol extraction method 72 hours after Transit-IT (Mirus)-mediated transfection of a pUASt-miR-1017 construct and Ubi-GAL4. cDNA was synthesized using Maxima H Minus Reverse Transcriptase and splicing patterns were observed for each of the constructed by PCR amplification of the region spanning miR-1017. Primers used to in this study for cloning constructs are provided in the Supplementary File 1.

### Lifespan analysis and climbing assay

For lifespan assessment, approximately 100 flies of each genotype were reared at 26°C, under a 12 h:12 h light:dark cycle. Groups of approximately 20 flies were collected at eclosure and aged separately. Flies were transferred to fresh food vials every 4 days, and the numbers of dead flies were counted daily. In climbing assays, 40 flies of each genotype were placed in an empty plastic vial. Flies were tapped to the bottom and the number of flies within the top, middle, or bottom of the vial were scored after 15 s. Experiments were repeated five times. For induction of geneswitch expression, mifepristone (ACROS organics) was dissolved in 100% ethanol and added at a final concentration of 100 μM per gram of standard fly food. Food was stored overnight to ensure even distribution of the drug before use. Additionally for antagonizing AchR activity, mecamylamine (ACROS organics) was dissolved in water and added at a final concentration of 10 μM per gram of standard fly food.

### RNA sequencing and computational analysis

Total RNA was extracted from the heads of w^1118^ or mutant flies using Trizol extraction. Samples were shipped to Novogene for sequencing using the Illumina platform. Samples were first subjected to quality control followed by library construction with the illumina TruSeq non-stranded Library Preparation Kit based on polyA capture and Reverse Transcription. Sequencing used a PE150 protocol. For small RNA sequencing, libraries were constructed using the TruSeq Small RNA Library Preparation Kit, and single-end sequencing was performed to produce 50-bp reads (SE50) using the Illumina platform.

Targets of miR-1017 are predicted by TargetScanfly 7.2 which lists 376 transcripts containing 413 conserved sites and 59 poorly conserved sites in the 3′UTRregion (20). For sites in open-reading frames (ORFs) TargetScanfly ORFs predicts 30 conserved targets with total of 30 conserved sites and 10 poorly conserved sites in the ORF (21). Gene Ontology analysis was carried out using GO enrichment R script (22). The resulting data was mapped to the reference genome (*Drosophila melanogaster r6.49*) using STAR aligner, which provided transcript quantification and splice junction analysis. Differential expression used the DESeq2 bioconductor package and the ggplot2 for visualization of the results (23). For branchpoint identification, all possible lariats from the miR-1017 intron were generated using LaSSO (24). Next, Bowtie2 was used to align a publicly available *Drosophila* circular RNA library based on RNA from fly heads depleted of linear RNA removed via RNase R to the possible lariats (25). Lariat alignments were visualized by matching to the miR-1017 sequence using Clustal Omega. Intronic reads alignments quantified and normalized to library depth. Computational Pipeline is diagramed in supplemental materials (Supplementary Figure S2).

## Results

### miR-1017 interacts with in acetylcholine signaling

To define a role of miR-1017 in fly biology, we first characterized target genes predicted by TargetScanfly 7.2 (16). Of the 406 predicted transcripts 376 had binding sites in 3′ untranslated regions (UTRs) and 30 in open reading fames (ORFs). Gene Ontology (GO) analysis of miR-1017 targets showed enrichment for GO terms related to neurobiology such as synaptic signaling, post synaptic membrane, channel activity, signaling receptor activity and acetylcholine receptor activity (Figure 1A) (Supplementary Table S1). Intriguingly, miR-1017′s host gene is nAchRa2/Dα2 is a predicted target due to an 8mer site in the ORF which is conserved from *D. melanogaster* to *D.wilistoni* with sporadic loss of the element in intermediate fly species. Additionally, another nAchR, *Dα5*, has two 8 mer UTR sites conserved across Drosophilids with intact sequences even in *D. grimshawi*. To test if miR-1017 can target these sites, luciferase sensors were generated. From co-transfection experiments in S2 cells both were significantly silenced compared to controls where the sequence was mutated (Figure 1B). The Dα2 ORF sensor was supressed 5-fold, the *Dα5* 3′UTR sensor 2-fold, and previously published Yan 3′UTR sensor 3-Fold (10) (Figure 1C).

**Figure 2.**
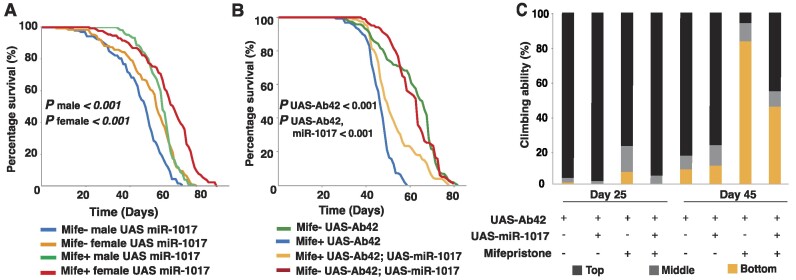
Ectopically expressed miR-1017 can prolong lifespan of *Drosophila melanogaster*. (**A**) The ectopic expression of miR-1017 under the ELAV-GAL4 driver induced with mifepristone revealed increased life span of wildtype male and female flies. Cox proportional hazard ratio test comparing mifepristone induced miR-1017 expression with the wildtypes (without ectopic expression) shows the *P* value <0.001 for both males and females. (**B**) Induced AD fly model lifespan presents a sharply reduced lifespan compared to the uninduced AD fly model. Ectopically driven miR-1017 within an AD fly model significantly prolongs lifespan. Cox proportional hazard ratio test comparing induced AD fly model against induced Ectopic miR-1017 within the AD fly model presents a *P* value of <0.001. Comparison of the uninduced lifespan analysis was not significantly different, *P* value = 0.71. (**C**) Climbing assay analysis reveals that ectopically driven miR-1017 within the AD fly model can dampen the neurological deficits. At day 25, ANOVA analysis of the AD fly model presents a significantly different climbing ability compared to ectopic miR-1017 within the AD fly model, *P* value <0.01. At day 45, ANOVA analysis of the AD fly model climbing ability is significantly different compared to the ectopically driven miR-1017 within the AD fly model, *P* value <0.001. ANOVA analysis of uninduced AD fly models showed no difference in climbing ability, *P* value = 0.27.

Since Dα2, the miR-1017 host gene, is broadly expressed in the nervous system we investigated phenotypes following ectopic expression of a UAS-miR-1017 construct through a ELAV:Geneswitch:GAL4 driver. The geneswitch system was used to test adult animals where miR-1017 is most abundant and avoid developmental effects from ectopic expression in earlier stages (26). Induced animals showed no overt phenotype, however, through monitoring lifespan we found ectopic expression of miR-1017 extended survival of both male and female flies by about ∼10 days (Figure 2A). To verify a potential neuroprotective function of miR-1017 we tested ectopically expressed miR-1017 in an Alzheimer's disease (AD) fly model based on a UAS-Aβ42 transgene that produces Amyloid Beta with the pathogenic arctic mutation (E22G substitution) (27). Expression of UAS-Αβ42 via the ELAV:Geneswitch:GAL4 driver led to a decreased overall lifespan of 45–55 days. Consistent with results from wild type backgrounds, miR-1017 expression significantly improved survival of the AD fly model by ∼20 days (Figure 2B). We also tested if miR-1017 can protect against loss of motor function in AD flies. At 25 days post eclosure (dpe), the AD fly model exhibited a slight reduction in climbing ability with approximately 20% of flies showing neurological deficits which were alleviated by co-expression of miR-1017. By day 45, the AD fly model showed severe neurological deficits, with 94% of flies performing poorly in the climbing assay but only 55% after miR-1017 was also expressed (Figure 2C). The ability of miR-1017 to reduce AD pathology is consistent with modulation of Ach signaling. One of the prominent features of AD is increased firing of background action potentials and neurotransmitter dysregulation (28). As Ach is the major excitatory neurotransmitter in Drosophila, suppression of receptor expression by miR-1017 could dampen this effect.

### Loss of miR-1017 function

**Figure 3.**
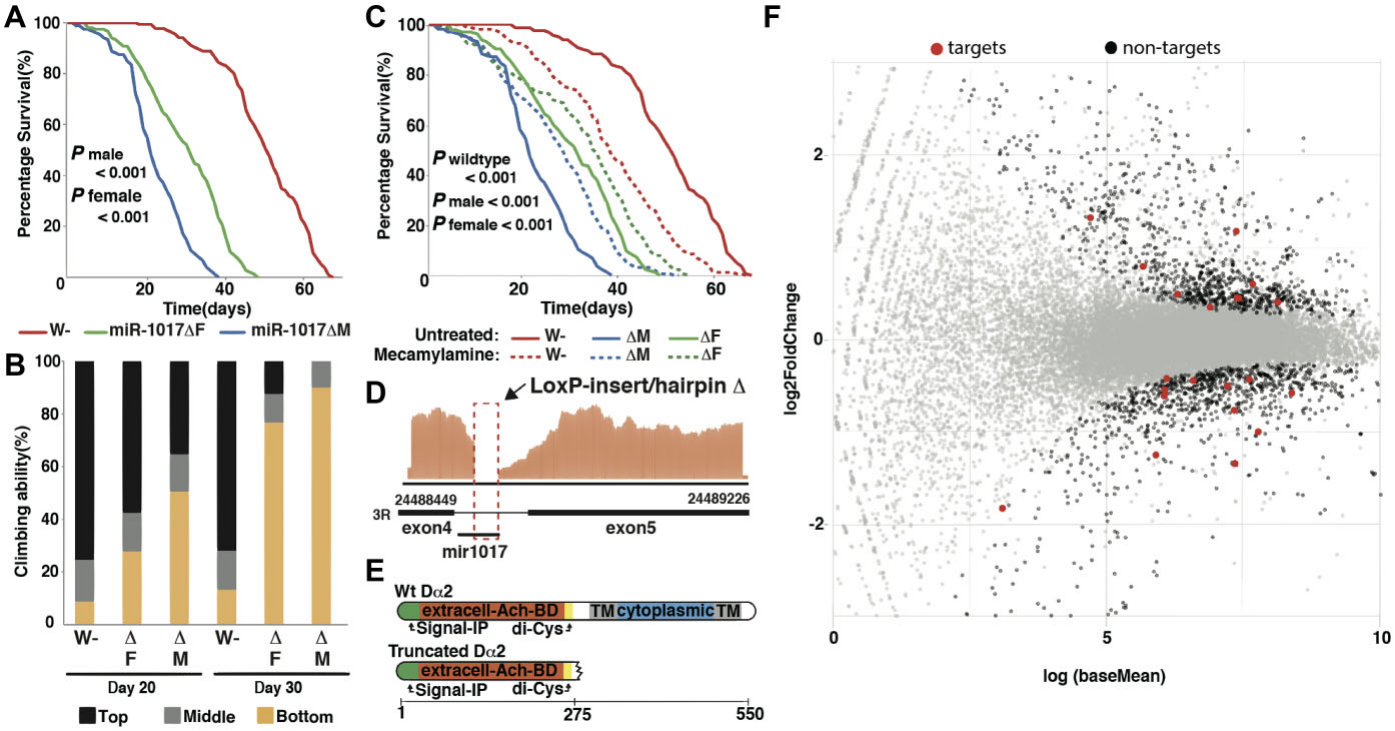
Deletion of miR-1017 impacts AchR signaling through abrogating splicing and generating truncated Dα2 protein. (**A**) Lifespan analysis reveals a greatly reduced lifespan for miR-1017Δ males and females, compared to wildtype populations. Cox proportional hazard ratio test: *P* value <0.001 comparing w- against miR-1017Δ male and female. (**B**) Climbing assay analysis presents neurological deficits at both day 20 and 30 for male and female miR-1017Δ compared to w-. ANOVA analysis presents significantly different climbing ability for male miR-1017Δ compared to w- at day 20 and 30, *P* value <0.001. Female miR-1017D are significantly different compared to w- at days 20 and 30 presents *P* value <0.05 and <0.001 respectively. Male miR-1017Δ compared against female miR-1017Δ present significantly different climbing ability at both day 20 and 30, *P* value <0.01. (**C**) life span of male (M) and female (F) miR-1017Δ flies after mecamylamine treatment as compared to untreated mutant flies. Cox proportional hazard ratio test comparing treated and untreated mutants (both male and female) shows *P*value <0.001. Treated w- flies were seen to have reduced life when compared to the untreated ones. (**D**) Sashimiplot was generated using alignment from miR-1017Δ flies. Site of indel indicated by red box. No junction reads were observed in alignments. The intron reads are normalized with the number of alignments in exon 4. (**E**) Diagram showing domains in Dα2 and those lost by a premature stop codon caused by failure to splice the miR-1017 exon. (**F**) Scatterplot generated using DESeq showing differentially expressed target and non-target genes in mutant flies as compared to the wildtype. grey dot denotes the genes that are not significant (*P* value > 0.05), red represent the targets genes of mir-1017 that are significant (*P* value < 0.05) and black represent significant non-target genes (*P* value < 0.05).

Next, we examined whether miR-1017 loss of function yielded phenotypes related to over expression. For this we obtained a publicly available miR-1017 deletion (miR-1017Δ) from the Bloomington Drosophila Stock center (29). This mutant is homozygous viable, exhibiting no overt morphological phenotype, however, animals exhibit lethargy suggesting a neurological phenotype. This is supported by mir-1017Δ survival declining rapidly between days 20–30, which is more severe in males (Figure 3A). miR-1017Δ flies also exhibit neuromuscular deficits whereby day 20, 50–65% of male mutants and 40–50% female mutants were impaired in their performance in a climbing assay. By day 30 both mutants exhibit severe neurological deficits with males (100%) and females (87.5%) impaired climbing (Figure 3B). As miR-1017 may regulate Ach signalling we sought to rescue the mir-1017Δ phenotype with the AchR antagonist mecamylamine (30). Treatment with mecamylamine significantly improved life span of both miR-1017Δ males and females. The opposite effect is seen in wildtype flies where treatment causes a significant reduction in lifespan, suggesting AchR activity was central to the reduced lifespan (Figure 3C). The reduced lifespan of wildtype flies following treatment is likely the effect of reduced acetylcholine signaling due to the drug's activity.

To better understand the molecular basis of the mir-1017Δ phenotype we performed RNA sequencing of heads to compare young (2 dpe) mutant and wildtype flies as miR-1017 expression peaks early in adults. Unexpectedly, from this data we discovered the lesion deleting miR-1017, which includes a ∼100 nt intact loxP site placed within the intron, leads to constitutive retention of the miR-1017 host intron (29) (Figure 3D). This is despite most of the intron elements remaining intact such as the 5′ splice site, 3′ splice site, and a strong poly-pyrimidine tract (ppt) sequence. Retention of the mutant intron leads to a premature stop codon that truncates the protein leaving an intact ligand binding domain while omitting the transmembrane portion of this Ach Receptor (Figure 3E). Such a mutation should lead to loss of Dα2 function as the intercellular signaling domain is absent, however, a N-term signal peptide would remain, suggesting deposition of the truncated protein in the ER and possible secretion. A di-cysteine motif is also retained in the truncated protein, which is essential for Ach binding. Treatment with mecamylamine rescues this phenotype, perhaps through interacting with the truncated protein that retains an intact Ach binding site and prevent it from interfering with other AchRs or the neurotransmitter itself.

Differentially expression of genes in miR-1017Δ compared to w^1118^ flies found very few predicted targets genes of miR-1017 significantly changed (Figure 3F). Out of 406 targets, only 21 showed a P value < 0.05 (Supplementary File 2). Out of the 21, 12 were downregulated and only 9 target genes were seen to be upregulated, which is inconsistent with loss of repression by miR-1017. Thus, loss of full-length Dα2 perturbed gene expression such that a distinct function of miR-1017 could not be discerned amid wider dysregulation. This is further evident from the failure to recover any of the GO terms enriched for targets of this miRNA (Supplementary Figure S3).

**Figure 4.**
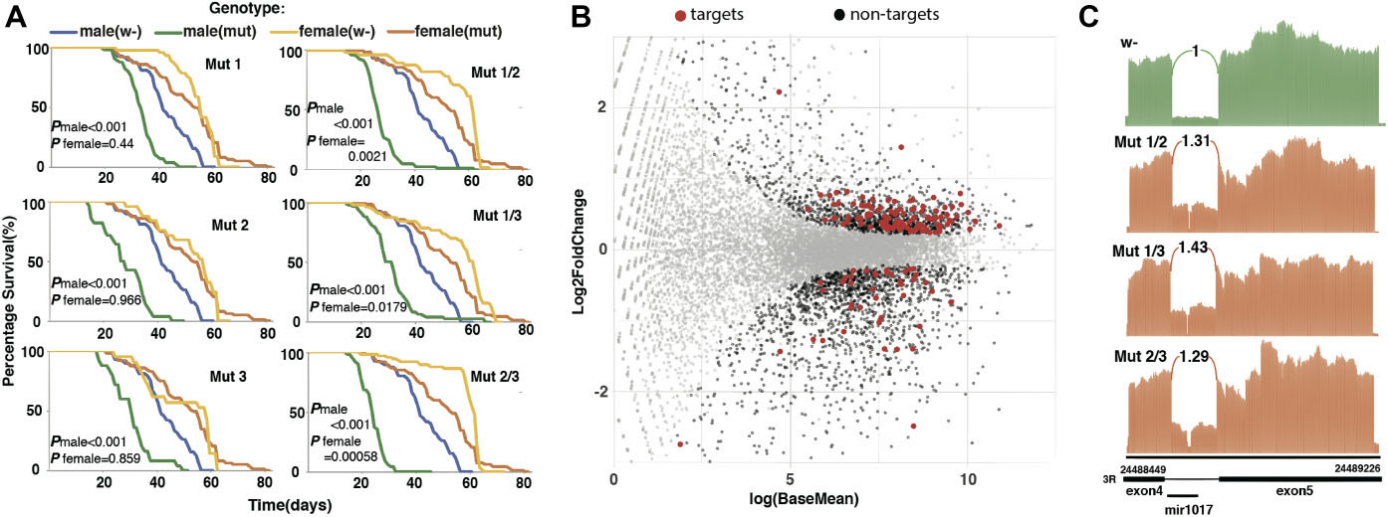
miR-1017 loss of function mutations reduces lifespan in males and impact splicing. (**A**) Lifespan analysis of wildtype and CRISPR mutant flies. 3 pure line mutants are represented as mut1, mut2 and mut3 respectively, while the cross lines are represented by mut 2–1, mut2-3 and mut1-3. mut2-1 denotes the cross line of mut 1 and 2 and so on. There was a significant reduction seen in genotypes of mutant in male flies all *P*-value being <0.01. However, there was no significant reduction seen in case of females (*P* value > 0.5). (**B**) Scatterplot showing differentially expressed target and non-target genes in mutants as compared to wildtype flies. Grey denotes genes that are not differentially expressed(*P*value > 0.05), red represent significant target genes and black represent significant non-target genes(*P*value < 0.05). (**C**) Sashimi plot generated using alignments files for wildtype (denoted by green) and mutant (denoted by orange) libraries. The numbers shown are the normalized intronic reads of mutants as compared to the wildtype which are first normalized with the number of alignments in exon 4 and then to the wildtype libraries. Mutant 2–1, 2–3 and 1–3 showed modestly higher, i.e 1.31×, 1.43× and 1.29× intron retention as compared to wildtype flies.

To identify a phenotype specifically associated with loss of miR-1017, we used the CRISPR/Cas9 system to generate three miR-1017 mutant fly lines. Using a PAM site in the miR-1017 hairpin loop, lesions were induced in the mature guide miRNA sequence (3′ arm) (Supplementary Figure S4A). The mutations occurred outside of the ‘seed’ element but altered the hairpin structure. Small RNA sequencing of mutant lines recovered no miRNAs produced from these hairpins; thus, mutations lead to abrogation of Dicer processing (Supplementary Figure S4B). Next to reduce the likelihood of background effects, trans-heterozygotes were generated by crossing lines in different combinations. We then carried out lifespan analysis of 6 mutant lines (3 homozygous and 3 trans-heterozygous). The results showed reduced male lifespan for all genotypes with all *P* values <0.05. This phenotype was not observed in females for homozygous lines (Figure 4A).

Next Total mRNA was sequenced from heads of w- and trans-heterozygous miR-1017 mutant male flies to assess differential regulation of targets. Of the 406 predicted targets, 151 were differentially expressed (with *P* value < 0.05) with 110 upregulated and only 41 down, which is more in line with loss of miRNA function (Figure 4B) (Supplementary File 3). Included in the upregulated targets were Dα2, Dα5 and two other AchRs (Dα3 and mAchR). Gene Ontology enrichment analysis the 1987 differentially expressed, upregulated genes found terms such as neuron projection, ion channel activity, and neurotransmitter receptor indicating miR-1017 significantly influences nervous system function (Supplementary Figure S5). To confirm differential expression from RNA sequencing, expression of five target genes of miR-1017 (Dα2, Dα5, aop E (spl)mgamma-HLH and notopleural) and a non-target gene quick-to-court (qtc) were assessed by RT-qPCR. The qPCR results shows that the target genes are significantly upregulated while the non-target gene was not (Supplementary Figure S6). This taken with the reduced lifespan phenotype potentially indicates that miR-1017 does appreciably influences *Drosophila* neurophysiology as a *trans-*regulator.

### miR-1017 host intron splicing

**Figure 5.**
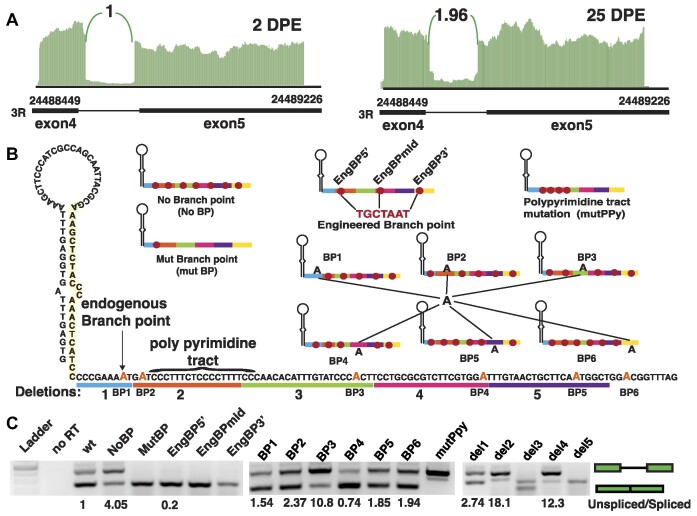
Control miR-1017 host intron splicing. (**A**) Sashimi plots of the miR-1017 host intron from RNA sequencing from DPE 2 and DPE 25 fly heads. Intronic reads were first normalized with the number of alignments in exon 4 and then to the wildtype libraries. (**B**) Identification and mutation of sequence elements in the miR-1017 host intron. The full sequence of the intron with the 5′ hairpin is shown across left and bottom. The endogenous branchpoint and strong pyrimidine tract are labelled. Deletions are shown by colored bars below. Right and above, are diagrams showing the mutant sequences made targeting Adenosine residues that could serve as branchpoints. (**C**) Each construct was expressed in S2 cells followed by RNA isolation and RT-PCR. Through size separation of products, the ratio of spliced and unspliced products were calculated.

As part of the RNA sequencing analysis from miR-1017 trans-heterozygote mutants, splicing of the host intron was examined. Even though only 2–4 nt residues were changed in mutants, modest intron retention could be seen compared to the wildtype flies (Figure 4C). Examining sequence elements potentially disrupted by the mutations found changes in a SRp55 binding site, which appears to be part of an intronic splicing enhancer (ISE) were several other SR proteins bind (31) (Supplementary Figure S7A). Despite the partial loss of this ISE, the degree of retention was not expected to lead to measurable loss of full-length Dα2, which could be seen from examining the number of splice junction reads that gapped over the miR-1017 intron (Supplementary Figure S7B). Spurred by this observation we sought other conditions where there was increased retention, which we found in aging flies, suggesting failure to splice is an intrinsic characteristic of the miR-1017 intron (Figure 5A). Like the homologous recombination insertion (miR-1017Δ) when the wildtype intron is retained a premature stop codon is introduced, leading to a nearly identical truncated protein.

To further examine the impact of the hairpin on splicing we tested several mutations of the structure (Supplementary Figure S9). First, we deleted conserved sequences in the hairpin (del-hpArms) that are not adjacent to the 5′ splice site (Supplementary Figure S10). Despite removal of these sequences which do contain an intronic splicing enhancer, no change in splicing was observed. This indicates the structure likely compromises the ISE as splicing is not affect when the both the structure and the element are removed. To dissect the significance of the structure from the ISE we replaced the miR-1017 hairpin with an alternative hairpin structure (hp-swap). This change completely eliminated splicing, thus with both a strong structure and loss of the ISE, splicing does not occur. Splicing could be partially restored by reverting the residues proximal to the 5′ splice site, which allows splice residues to remain unpaired (hp-swap-wt5ss). This supports the hypothesis that the hairpin adjacent to the splice site impacts splicing of this intron, and that adding unpaired residues juxtaposed to the splice site leads to more reliable recognition by U1.

As minor changes in the miR-1017 hairpin impacts splicing, we sought to further understand sequence elements that guide splicing of this intron. Due to poor definition of the intron despite consensus 5′ and 3′ splice sites, we focused on contribution of the branch point to increased retention. Using circularized RNA libraries and a virtual sequence library of several thousand possible lariat branch point locations generated by laSSO, a verified branch point was found 8bp from the hairpin (Figure 5B) (Supplementary Figure S8) (24). Next, we generated a variety of mutations in a minigene construct to explore branch point behavior in an ectopic expression system in S2 cells. Following transfection, spliced to unspliced ratio could be assessed by RT-PCR (Figure 5C). First, we tested the consequence of removing the endogenous branch point (MutBP) and found reduced retention compared to wildtype. This suggests that choice of another adenine residue in the tail or hairpin could act as branch point and function with greater fidelity. Removing all adenosine residues from the tail (NoBP) and forcing branch point use in the hairpin shows accurate splicing but also 4x increased intron retention. Beginning with this construct we inserted a consensus branch point element at three positions within the tail (EngBP5’, EngBPmid and EngBP3’). All three restored full splicing, with very little retention. The only construct that showed retention was EngBP5’, which is the closest to the endogenous splice site. Next, again starting with the NoBP construct, six individual adenosine residues were restored in the intron (BP1-6). Restoration of the endogenous branchpoint (BP1) improved splicing, but not to wild type levels, indicating loss of most adenosines in the tail alone impairs splicing by itself. Reversal of other adenosines led to inconsistent restoration of splicing. Most notable was BP3 which led to significant increase in retention, potentially due to aborted splicing. This contrasts with BP4 which improved splicing fidelity over wildtype. The difference in BP3 and BP4 appears to be proximity to a secondary polypyrimidine (ppy) tract, which led us to generate an additional construct deleting this element. Loss of the primary ppy tract and all adenosines completely abolished splicing.

We then deleted different segments of the intron to test the importance of elements they contain (Figure 5C). Deletion of the hairpin base (del1) increased retention potentially by eliminating all but one adenosine between the ppy tract and the hairpin base. Expectedly, eliminating most of the ppy element in del2 greatly disturbed splicing. Interestingly, del 3 led to no unspliced transcripts and potential usage of a downstream cryptic 3′ splice site. This contrasted with del4 which greatly impairs splicing. These results are potentially a result of consolidating pyrimidine rich aspects of the intron. Del4 contains a secondary ppy tract, which might be competing with the stronger ppy upstream. In del5, bringing the 3′ splice site closer to the weaker ppy tract leads to no unspliced products. Together these results indicate that retention is due to interplay between different elements that can serve as a ppy tract and their role in defining the branch. It also appears the stronger ppy tract is compromised by the proximity to the hairpin. Indeed, the modest changes in the hairpin introduced by genome editing impact intron definition, likely through affecting branchpoint function. Thus, the miR-1017 hairpin not only is able to generate *trans*-acting regulators through miRNA production, but also influences splicing of the host intron.

## Discussion

This work shows a role for miR-1017 in regulating *Drosophila* neurobiology genes, including its host gene, Dα2 (Figure 1). This is the second study to report a phenotype associated with mirtron loss of function and the first to define the roll of a ‘tailed’ mirtron subclass. The major function of miR-1017 is to promote longer lifespan in *Drosophila*. Overexpression of miR-1017 extends the life span of wildtype flies and can even rescue an early death phenotype in an Alzheimer's model. (Figure 2A-C). Loss of miR-1017 function leads to an opposite phenotype where flies, particularly males, exhibit a reduced lifespan (Figures 3A and 4A). This work also found the structure of the miR-1017 hairpin has significance beyond being a Dicer substrate and source of small RNAs. The hairpin impacts splicing of the host intron, which is prone to retention (Figure 4C). This suggests a model where mirtron evolution is connected to not only target regulation but also to host transcript processing.

Mirtrons are defined by their proximity to splice sites and their hairpin structures can be expected to impact binding of U1, U2 and U2AF (32). Indeed, structures encompassing 5′ splice sites and branchpoints have been found to inhibit splicing by affecting recruitment of U1 and U2. Alternatively, structures can also enhance fidelity by bringing splicing elements (i.e. 3′splice site and branch point) closer (33). In case of miR-1017, 5′ splice site residues are paired in the base of the hairpin, which likely restricts binding by U1 and may explain why retention is seen for the intron. Further, near the 3′ arm of the hairpin is the branchpoint which could also block snRNP binding. In addition to the compromised 5′ splice site and branchpoint elements, the hairpin also seems to impact recognition of the ppy tract, as two potential tracts are present in the intron. The one closest to the hairpin is a perfect consensus element with 20 consecutive pyrimidine residues where the more 3′ element is interrupted by several guanosine residues. The overwhelming strength of the first element likely biases branchpoint usage to the residue juxtaposed to the hairpin. Interestingly, in other Drosophilids the ppy tract is in the same region of the intron, suggesting there is some conservation of the branchpoint placement and a possible benefit to defective splicing. However, completely replacing the hairpin sequence with a similarly constituted structure leads to loss of intron recognition (Supplementary Figure S9). This appears to be due to the presence of an ISE in the 3′ hairpin arm (Supplementary Figures S7A and 10). Thus, there is likely also selective pressure on the precise sequence in addition to the structure.

Conservation of poor definition of the miR-1017 intron may be connected to nature of Dα2. Multiple studies have reported intron retention in insect acetylcholine receptors (34,35). The most striking example in *melanogaster* is the abundant incompletely spliced Dβ1 transcripts that alter the temporal expression of fully spliced mRNA isoforms (36). Further, an alternatively spliced isoform of Dα4 has been shown to greatly impact the nicotinic receptor assembly leading to reduced nicotine binding (34). Thus, regulation of acetylcholine receptor function by modulating RNA processing seems to be common phenomenon in *Drosophila* and other invertebrates. In the case of Dα2, retention of the miR-1017 intron would lead to truncation where only the extracellular domain would be translated. This opens the possibility that the truncated protein instead of being part of the receptor complex could act as a secreted protein which could still bind acetylcholine in the extracellular space and alter signaling. The truncated isoform is not explicitly toxic as we observed viability in the miR-1017 recombination mutants where splicing is abrogated. Part of miR-1017 hairpin conservation may be to compromise splice elements, and may explain the origin of the structure, which then later acquired *trans*-acting function through becoming a mirtron. Targeting of Dα2 by miR-1017 in *trans* would strengthen the regulatory impact of the intron on host gene function.

On a broad level, our results suggest a mechanism for the birth of mirtrons. In most cases, mirtrons are young genes that tend to initially evolve almost neutrally with many rapidly lost from genomes (37). This is consistent with a process where initial events in their evolution are driven by structure and not explicitly sequence. As structures become suitable Dicer substrates, they may occasionally integrate into gene regulatory networks, gaining function and then evolving more slowly, becoming conserved at the sequence level in multiple genomes. However, even these ‘functional’ mirtrons (i.e. miR-1003, miR-1004 and miR-1010 *in Drosophila*, or mir-887 and miR-1224 in mammals) aren’t maintained outside related groups. Poor conservation of mirtrons cannot be explicitly linked to their expression being coupled with a mRNA as there are many canonical miRNAs found in introns. However, our data shows mirtrons differ from canonical intronic miRNAs as they directly interact with splicing elements, and that it may be the need for genes to innovate at the level of splicing regulation that leads to loss of mirtrons. This is consistent with identification of mirtrons associated with more complex tissues like the nervous system where fine tuning of gene expression is critical. For example, multiple non-canonical miRNAs including mirtrons were found to be highly expressed in the hippocampus and cortex relative to other tissues of mice (38). In another study 16 primates specific mirtrons including some tailed mirtrons were confidently characterized from human brain (39). modENCODE expression data of mirtron's host genes in *D. melanogaster* revealed that they had significant expression in nervous system (40).

Together our results find that a *Drosophila* 3′-tailed mirtron, miR-1017, has a measurable phenotype that manifests as promoting an extended lifespan. Through this work we also find that the precursor interacts with splicing regulation of the intron. This discovery led us to propose a mechanism for mirtron birth and death being potentially tied to splicing regulation. In this model, control of splicing would have precedence over *trans*-regulatory function of the mirtron, which may explain their short-lived evolutionary histories. Additional studies will need to explore the interaction of mirtrons with other splicing elements and the order events in terms of hairpin folding versus snRNP binding. Another outstanding question is how the abundant 5′-tailed mirtrons in mammals may integrate with regulatory mechanisms that modulate recognition of 3′ splice site elements. It also suggests a similar situation could be occurring for other non-canonical miRNAs where structural constraints outside the need to create a Dicer substrate influence conservation.

## Supplementary Material

gkad1158_supplemental_files

## Data Availability

The data resulting from High throughput sequencing (fastq files) has been deposited in the Sequence Read Archive (SRA) repository under accession number PRJNA948397.

## References

[1] Flynt A.S. , Lai E.C. Biological principles of microRNA-mediated regulation: shared themes amid diversity. Nat. Rev. Genet. 2008; 9 831–842. 18852696 10.1038/nrg2455 PMC2729318

[2] Guo H. , Ingolia N.T., Weissman J.S., Bartel D.P. Mammalian microRNAs predominantly act to decrease target mRNA levels. Nature. 2010; 466:835–840. 20703300 10.1038/nature09267 PMC2990499

[3] Bartel D.P. MicroRNAs: genomics, biogenesis, mechanism, and function. Cell. 2004; 116:281–297. 14744438 10.1016/s0092-8674(04)00045-5

[4] Lee Y. , Kim M., Han J., Yeom K.H., Lee S., Baek S.H., Kim V.N. MicroRNA genes are transcribed by RNA polymerase II. EMBO J. 2004; 23:4051–4060. 15372072 10.1038/sj.emboj.7600385 PMC524334

[5] Cifuentes D. , Xue H., Taylor D.W., Patnode H., Mishima Y., Cheloufi S., Ma E., Mane S., Hannon G.J., Lawson N.D. et al. A novel miRNA processing pathway independent of dicer requires argonaute2 catalytic activity. Science. 2010; 328:1694–1698. 20448148 10.1126/science.1190809 PMC3093307

[6] Yang J.S. , Lai E.C. Dicer-independent, Ago2-mediated microRNA biogenesis in vertebrates. Cell Cycle. 2010; 9:4455–4460. 21088485 10.4161/cc.9.22.13958 PMC3048044

[7] Ruby J.G. , Jan C.H., Bartel D.P. Intronic microRNA precursors that bypass Drosha processing. Nature. 2007; 448:83–86. 17589500 10.1038/nature05983 PMC2475599

[8] Okamura K. , Hagen J.W., Duan H., Tyler D.M., Lai E.C. The mirtron pathway generates microRNA-class regulatory RNAs in Drosophila. Cell. 2007; 130:89–100. 17599402 10.1016/j.cell.2007.06.028 PMC2729315

[9] Westholm J.O. , Lai E.C. Mirtrons: microRNA biogenesis via splicing. Biochimie. 2011; 93:1897–1904. 21712066 10.1016/j.biochi.2011.06.017 PMC3185189

[10] Flynt A.S. , Greimann J.C., Chung W.J., Lima C.D., Lai E.C. MicroRNA biogenesis via splicing and exosome-mediated trimming in Drosophila. Mol. Cell. 2010; 38:900–907. 20620959 10.1016/j.molcel.2010.06.014 PMC2904328

[11] Wen J. , Ladewig E., Shenker S., Mohammed J., Lai E.C. Analysis of nearly one thousand mammalian mirtrons reveals novel features of dicer substrates. PLoS Comput. Biol. 2015; 11:e1004441. 26325366 10.1371/journal.pcbi.1004441 PMC4556696

[12] Sibley C.R. , Seow Y., Saayman S., Dijkstra K.K., El Andaloussi S., Weinberg M.S., Wood M.J.A. The biogenesis and characterization of mammalian microRNAs of mirtron origin. Nucleic Acids Res. 2012; 40:438–448. 21914725 10.1093/nar/gkr722 PMC3245937

[13] Kock K.H. , Kong K.W., Hoon S., Seow Y. Functional VEGFA knockdown with artificial 3′-tailed mirtrons defined by 5′ splice site and branch point. Nucleic Acids Res. 2015; 43:6568–6578. 26089392 10.1093/nar/gkv617 PMC4513878

[14] Amourda C. , Saunders T.E. The mirtron miR-1010 functions in concert with its host gene SKIP to balance elevation of nAcRβ2. Sci. Rep. 2020; 10:1688. 32015391 10.1038/s41598-020-58655-7 PMC6997181

[15] Da Fonseca B.H.R. , Domingues D.S., Paschoal A.R. mirtronDB: a mirtron knowledge base. Bioinformatics. 2019; 35:3873–3874. 30874795 10.1093/bioinformatics/btz153 PMC6761972

[16] Agarwal V. , Subtelny A.O., Thiru P., Ulitsky I., Bartel D.P. Predicting microRNA targeting efficacy in Drosophila. Genome Biol. 2018; 19:152. 30286781 10.1186/s13059-018-1504-3 PMC6172730

[17] Bacher I. , Wu B., Shytle D.R., George T.P. Mecamylamine a nicotinic acetylcholine receptor antagonist with potential for the treatment of neuropsychiatric disorders. Expert Opin. Pharmacother. 2009; 10:2709–2721. 19874251 10.1517/14656560903329102

[18] Korona D. , Dirnberger B., Giachello C.N.G., Queiroz R.M.L., Popovic R., Müller K.H., Minde D.P., Deery M.J., Johnson G., Firth L.C. et al. Drosophila nicotinic acetylcholine receptor subunits and their native interactions with insecticidal peptide toxins. eLife. 2022; 11:e74322. 35575460 10.7554/eLife.74322 PMC9110030

[19] Chen Y.W. , Song S., Weng R., Verma P., Kugler J.M., Buescher M., Rouam S., Cohen S.M. Systematic study of Drosophila MicroRNA functions using a collection of targeted knockout mutations. Dev. Cell. 2014; 31:784–800. 25535920 10.1016/j.devcel.2014.11.029

[20] Friedman R.C. , Farh K.K.-H., Burge C.B., Bartel D.P. Most mammalian mRNAs are conserved targets of microRNAs. Genome Res. 2009; 19:92–105. 18955434 10.1101/gr.082701.108 PMC2612969

[21] Schnall-Levin M. , Zhao Y., Perrimon N., Berger B. Conserved microRNA targeting in Drosophila is as widespread in coding regions as in 3′UTRs. Proc. Natl. Acad. Sci. U.S.A. 2010; 07:15751–15756. 10.1073/pnas.1006172107 PMC2936641 20729470

[22] Alexa A. , Rahnenfuhrer J. topGO: enrichment analysis for gene ontology. 2023; Bioconductor 10.18129/B9.bioc.topGO.

[23] Love M.I. , Huber W., Anders S. Moderated estimation of fold change and dispersion for RNA-seq data with DESeq2. Genome Biol. 2014; 15:550. 25516281 10.1186/s13059-014-0550-8 PMC4302049

[24] Bitton D.A. , Rallis C., Jeffares D.C., Smith G.C., Chen Y.Y.C., Codlin S., Marguerat S., Bähler J. LaSSO, a strategy for genome-wide mapping of intronic lariats and branch points using RNA-seq. Genome Res. 2014; 24:1169–1179. 24709818 10.1101/gr.166819.113 PMC4079972

[25] Langmead B. , Salzberg S.L. Fast gapped-read alignment with Bowtie 2. Nat. Methods. 2012; 9:357–359. 22388286 10.1038/nmeth.1923 PMC3322381

[26] Ford D. , Hoe N., Landis G.N., Tozer K., Luu A., Bhole D., Badrinath A., Tower J. Alteration of Drosophila life span using conditional, tissue-specific expression of transgenes triggered by doxycyline or RU486/Mifepristone. Exp. Gerontol. 2007; 42:483–497. 17349761 10.1016/j.exger.2007.01.004 PMC1992522

[27] Iijima K. , Chiang H.C., Hearn S.A., Hakker I., Gatt A., Shenton C., Granger L., Leung A., Iijima-Ando K., Zhong Y. Aβ42 mutants with different aggregation profiles induce distinct pathologies in Drosophila. PLoS One. 2008; 3:e1703. 18301778 10.1371/journal.pone.0001703 PMC2250771

[28] Yasuyama K. , Salvaterra P.M. Localization of choline acetyltransferase-expressing neurons in Drosophila nervous system. Microsc. Res. Tech. 1999; 45:65–79. 10332725 10.1002/(SICI)1097-0029(19990415)45:2<65::AID-JEMT2>3.0.CO;2-0

[29] Chen Y.W. , Song S., Weng R., Verma P., Kugler J.M., Buescher M., Rouam S., Cohen S.M. Systematic study of Drosophila MicroRNA functions using a collection of targeted knockout mutations. Dev. Cell. 2014; 31:784–800. 25535920 10.1016/j.devcel.2014.11.029

[30] Bacher I. , Wu B., Shytle D.R., George T.P. Mecamylamine – a nicotinic acetylcholine receptor antagonist with potential for the treatment of neuropsychiatric disorders. Expert Opin. Pharmacother. 2009; 10:2709–2721. 19874251 10.1517/14656560903329102

[31] Cartegni L. , Wang J., Zhu Z., Zhang M.Q., Krainer A.R. ESEfinder: a web resource to identify exonic splicing enhancers. Nucleic Acids Res. 2003; 31:3568–3571. 12824367 10.1093/nar/gkg616 PMC169022

[32] Warf M.B. , Berglund J.A. Role of RNA structure in regulating pre-mRNA splicing. Trends Biochem. Sci. 2010; 35:169–178. 19959365 10.1016/j.tibs.2009.10.004 PMC2834840

[33] Chebli K. , Gattoni R., Schmitt P., Hildwein G., Stevenin J. The 216-nucleotide intron of the E1A pre-mRNA contains a hairpin structure that permits utilization of unusually distant branch acceptors. Mol. Cell. Biol. 1989; 9:4852–4861. 2601698 10.1128/mcb.9.11.4852-4861.1989 PMC363635

[34] Lansdell S.J. , Millar N.S. Cloning and heterologous expression of Dα4, a Drosophila neuronal nicotinic acetylcholine receptor subunit: identification of an alternative exon influencing the efficiency of subunit assembly. Neuropharmacology. 2000; 39:2604–2614. 11044730 10.1016/s0028-3908(00)00111-8

[35] Jin Y. , Tian N., Cao J., Liang J., Yang Z., Lv J. RNA editing and alternative splicing of the insect nAChR subunit alpha6 transcript: evolutionary conservation, divergence and regulation. BMC Evol. Biol. 2007; 7:98. 17597521 10.1186/1471-2148-7-98 PMC1919356

[36] Hermans-Borgmeyer I. , Hoffmeister S., Sawruk E., Betz H., Schmitt B., Gundelfinger E.D. Neuronal acetylcholine receptors in drosophila: mature and immature transcripts of the ard gene in the developing central nervous system. Neuron. 1989; 2:1147–1156. 2624744 10.1016/0896-6273(89)90181-5

[37] Butkytė S. , Čiupas L., Jakubauskienė E., Vilys L., Mocevicius P., Kanopka A., Vilkaitis G. Splicing-dependent expression of microRNAs of mirtron origin in human digestive and excretory system cancer cells. Clin. Epigenetics. 2016; 8:33. 27019673 10.1186/s13148-016-0200-y PMC4807562

[38] Babiarz J.E. , Hsu R., Melton C., Thomas M., Ullian E.M., Blelloch R. A role for noncanonical microRNAs in the mammalian brain revealed by phenotypic differences in Dgcr8 versus Dicer1 knockouts and small RNA sequencing. RNA. 2011; 17:1489–1501. 21712401 10.1261/rna.2442211 PMC3153973

[39] Berezikov E. , Chung W.J., Willis J., Cuppen E., Lai E.C. Mammalian mirtron genes. Mol. Cell. 2007; 28:328–336. 17964270 10.1016/j.molcel.2007.09.028 PMC2763384

[40] Li J.J. , Huang H., Bickel P.J., Brenner S.E. Comparison of D. melanogaster and C. elegans developmental stages, tissues, and cells by modENCODE RNA-seq data. Genome Res. 2014; 24:1086–1101. 24985912 10.1101/gr.170100.113 PMC4079965

