# Supplementary material for: A tailed mirtron promotes longevity in Drosophila
Source: Nucleic Acids Res. 2023 Dec 4;52(3):1080–9. doi: 10.1093/nar/gkad1158 (PMC10853799; doi:10.1093/nar/gkad1158)
Supplement: gkad1158_supplemental_files [file gkad1158_supplemental_files.zip › Khanal_supp_materials.docx]

**Supplementary Materials**

|  | **GO Terms** | **Expected** | **Observed** |
| --- | --- | --- | --- |
| Biological process | synaptic transmission | 15 | 4.93 |
|  | signaling | 52 | 31.11 |
|  | Cell communication | 52 | 31.43 |
| Cellular component | Plasma membrane | 34 | 19.7 |
|  | synapse | 16 | 6.99 |
|  | Post synaptic membrane | 6 | 1.13 |
| Molecular function | DNA transcription repressor | 9 | 1.61 |
|  | Channel activity | 3 | 0.14 |
|  | Signaling receptor activity | 15 | 5.92 |
|  | Acetylcholine receptor | 3 | 0.19 |

**Supplement Table 1**: GO enrichment of miR-1017 targets


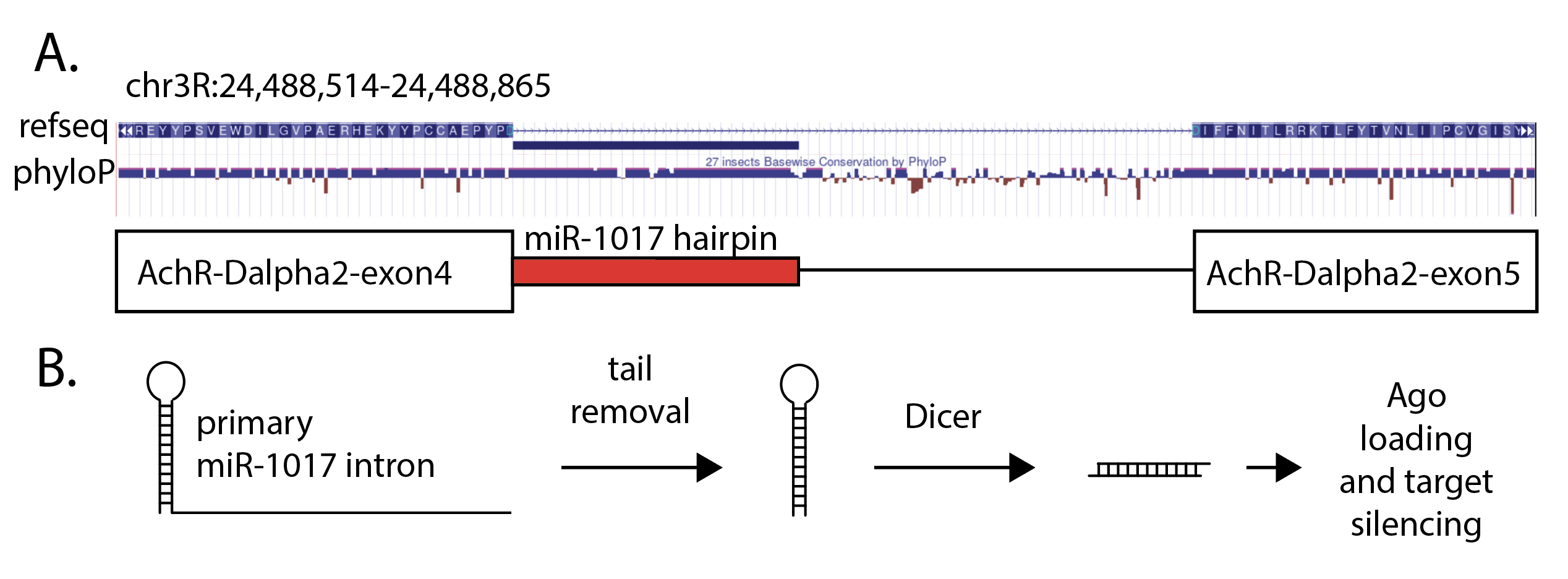


**Supplemental Figure 1: Diagram of the miR-1017 locus and biogenesis.** A. Genomic locus on chromosome three. Top track is from UCSC showing the codons in exons 4 and 5 of the AchR-Dalpha2 gene. The thick line below denotes the location of the miR-1017 hairpin. The phyloP line from UCSC shows conservation at the locus. Higher conservation is seen in the region encoding the hairpin. Below the UCSC tracks are a diagram showing the exons and miR-1017 exons. B. Simplified biogenesis of miR-1017 where after splicing the intron is trimmed to yield a hairpin that is a suitable Dicer substrate. After Dicer cleavage, miR-1017 small RNAs are loaded into Ago complexes and act as miRNAs.


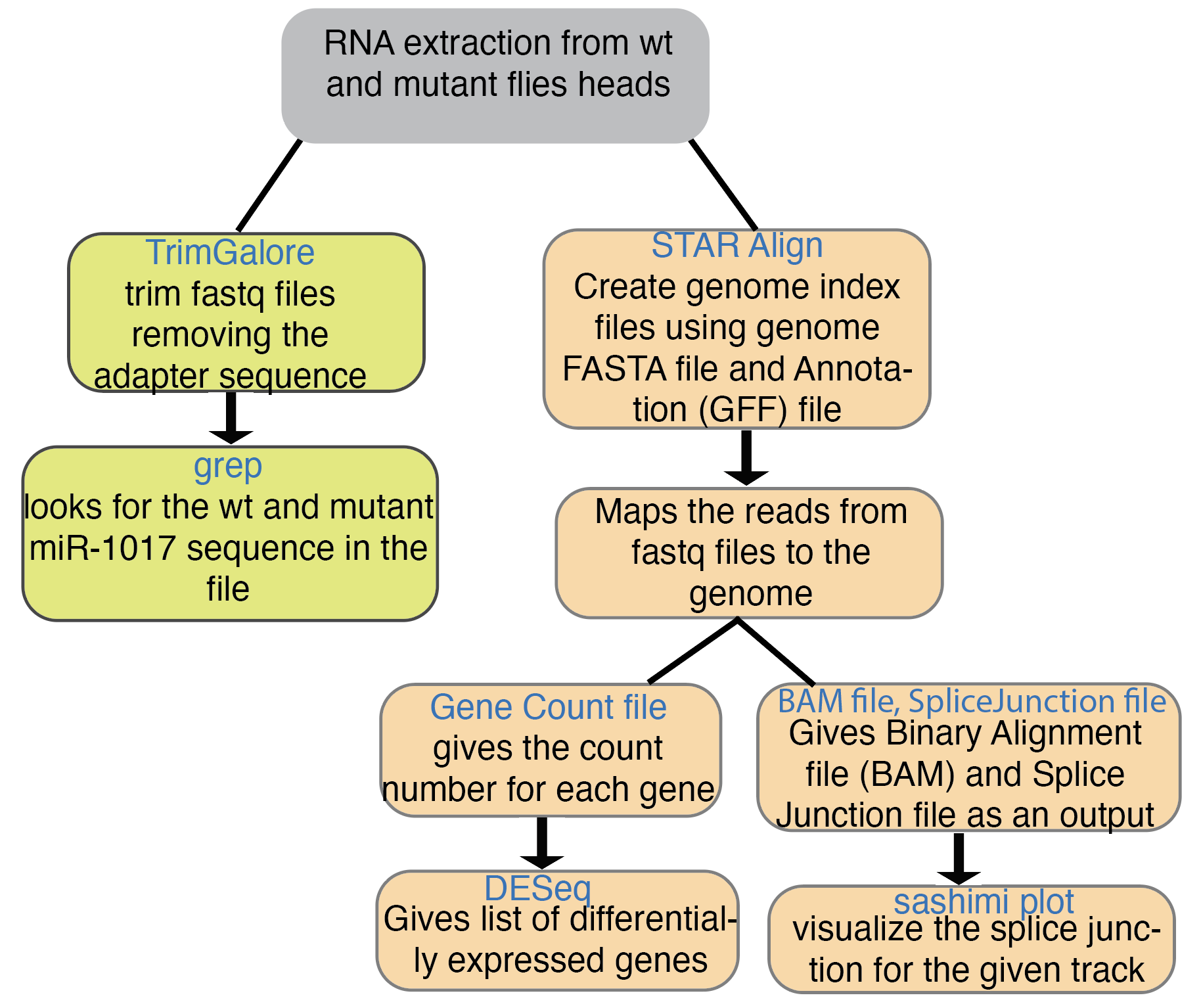


**Supplemental Figure2:** Computational pipeline

**Supplement Fig 3**: GO enrichment of upregulated transcripts in miR-1017 mutants generated by homologous recombination.

***
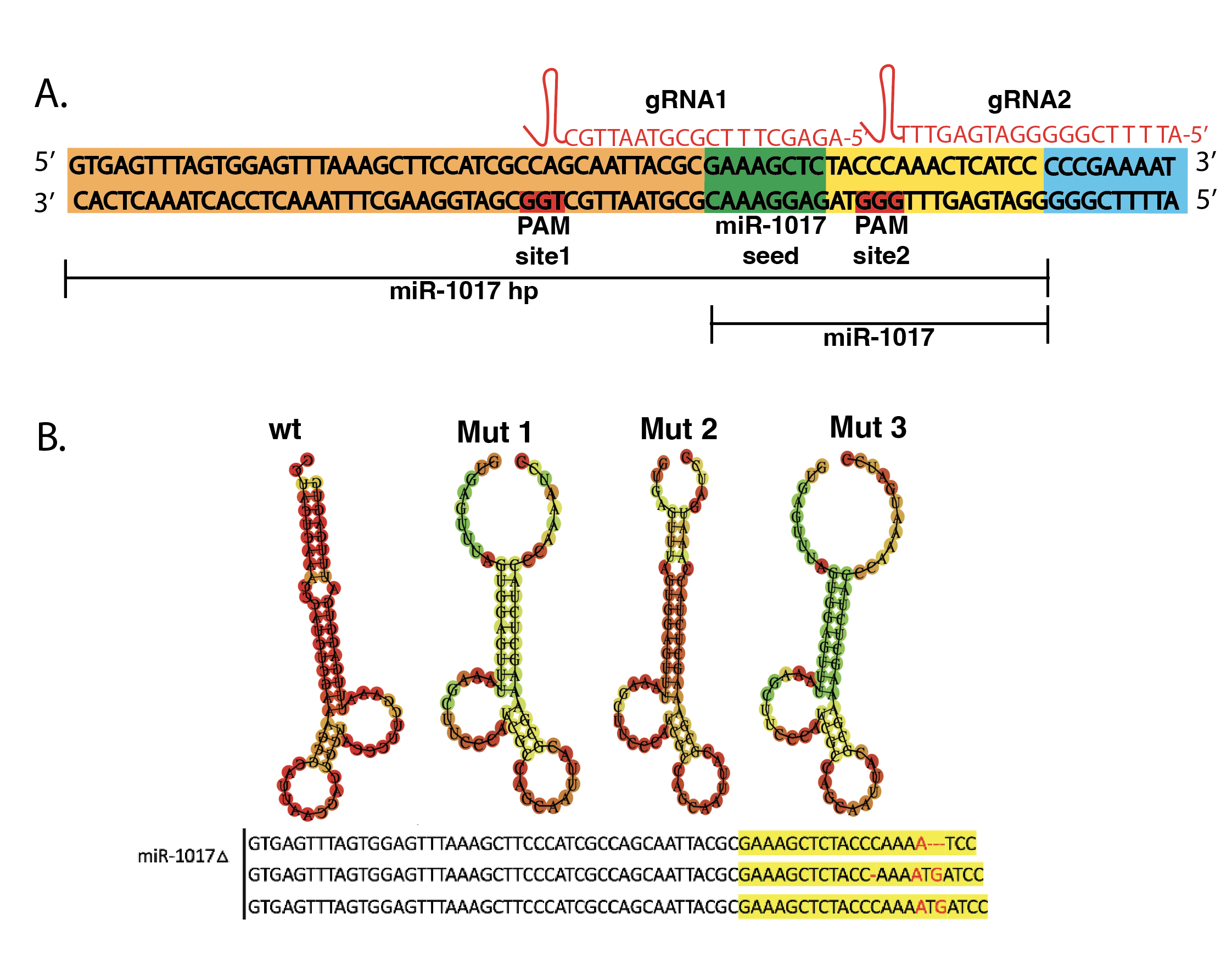
***

**Supplement Fig 4: Generation of miR-1017 mutation with gene editing and resulting structures** (A) Design of two guide RNAs (gRNA1 and gRNA2) targeting the mature miR-1017 sequence. (B) Hairpin structure of the mutant lines generated by RNA-fold as compared to the wildtype with SNPs and indels introduced in each of mutant lines mir-1017 sequence can be seen below with residues highlighted in red.

**Supplement Fig 5**: GO enrichment of upregulated transcripts in miR-1017 mutants created by CRISPR/Cas9

******

**Supplemental Fig 6: RT-qPCR of miR-1017 targets and non-target control.** RNAs were extracted from wildtype and mutant flies head 2 days post eclosure. Data are mean ± SD (n=3). Statistical analysis was performed with a two-tailed and unpaired Student t test. **p* < 0.05, ***p* < 0.01,****p* < 0.001 *****p* < 0.0001.


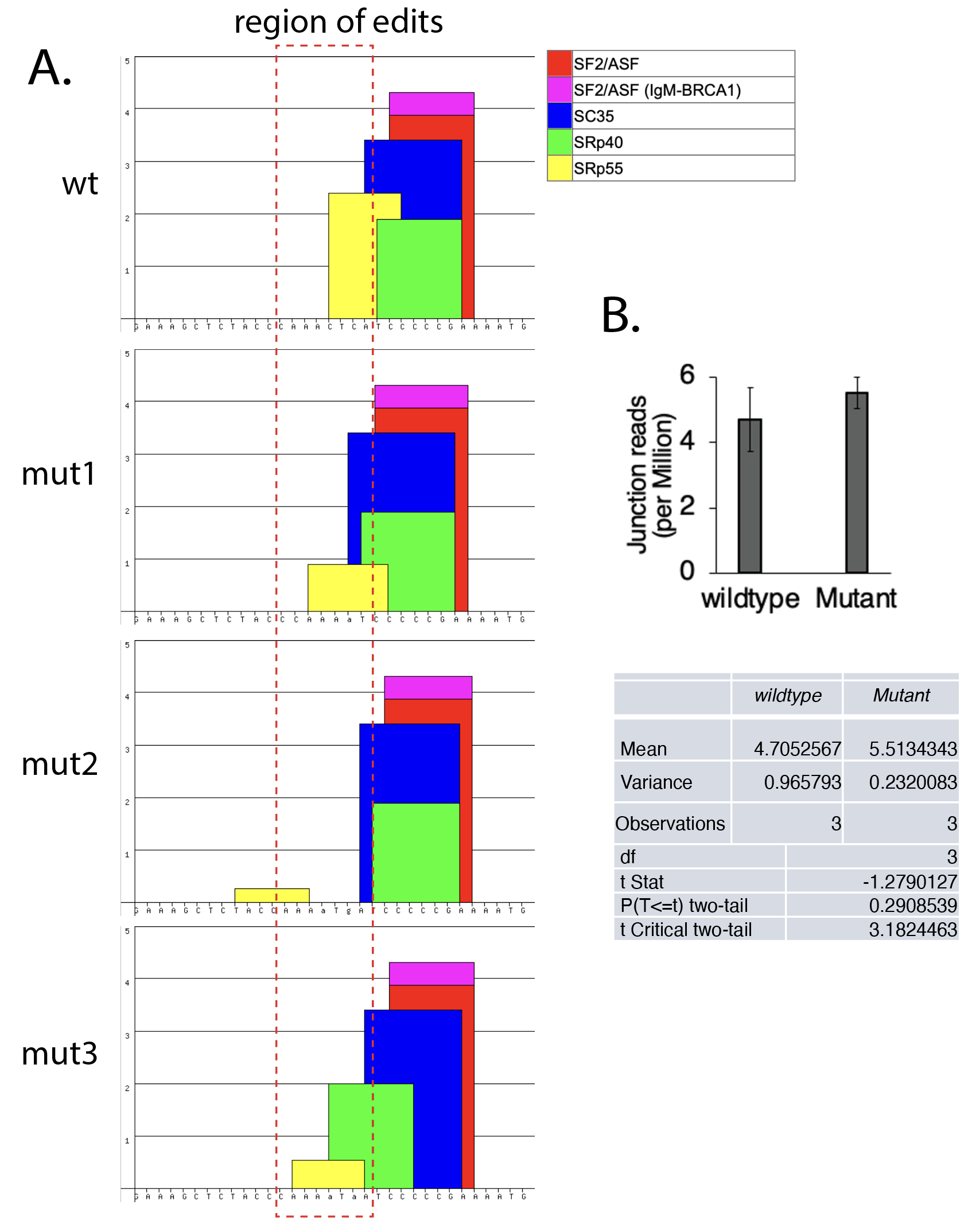


**Supplemental Figure 7: Impacts of genome edited mutants on splice elements and abundance of splice products (**A) In silico identification of SR protein binding determined by ESEfinder. Mutations caused by gene editing appear to affect an SRp55 binding site. Red, dashed-line box indicates location of edited bases. Legend on right shows the color-coded identity of SR proteins. (B) Junction reads spanning the miR-1017 intron in wildtype and CRISPR generated mutant flies. No significance difference is seen as p > 0.05.

***
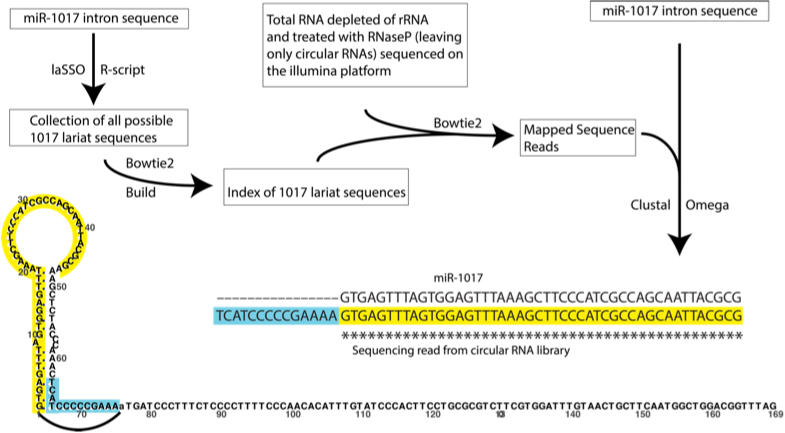
***

**Supplemental Fig 8**: Branch Point Identification of miR-1017

***
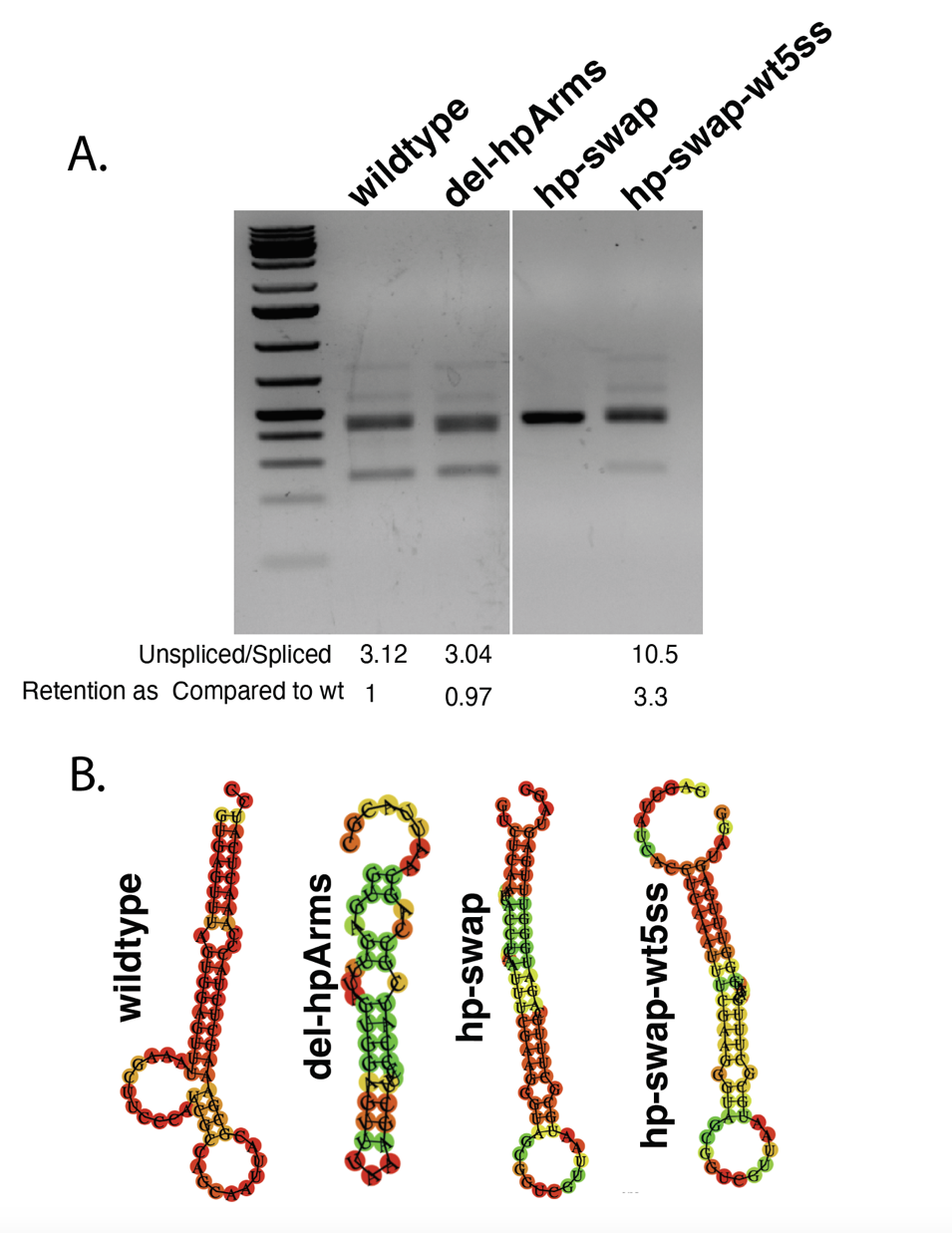
***

**Supplementary Figure 9: Mutations in the miR-1017 hairpin and impact on splicing** (A) RT-PCR of additional hairpin mutants. Constructs were cloned into pUastDsRed plasmid, transfected into S2 cells followed by RNA extraction and RT-PCR. Unspliced to spliced ratio of the PCR products was calculated (B) RNA fold of the hairpin constructs.


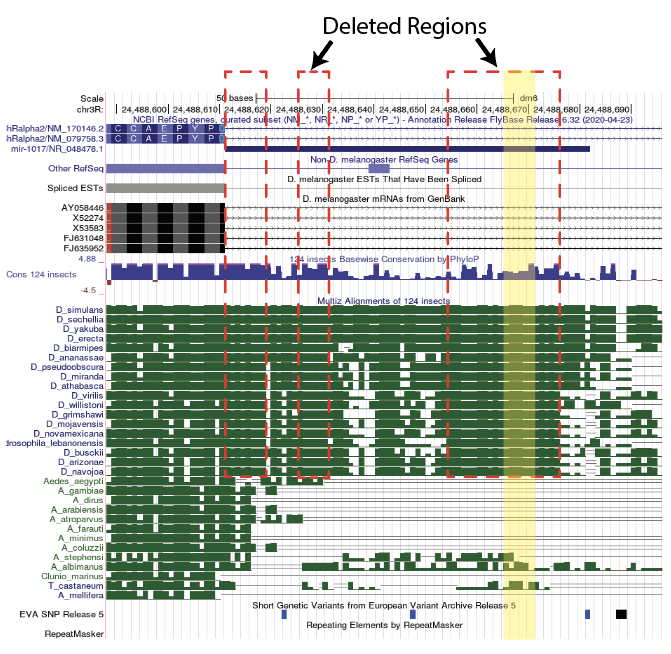


**Supplementary Figure 10:** Conserved features of miR-1017 shown in boxes with red dashed lines. Yellow highlight shows location of predicted Intronic Splicing Enhancer (ISE). The regions deleted in the del-hpArms constructs denoted.
