# Supplementary material for: A tailed mirtron promotes longevity in Drosophila
Source: Nucleic Acids Res. 2023 Dec 4;52(3):1080–9. doi: 10.1093/nar/gkad1158 (PMC10853799; doi:10.1093/nar/gkad1158)
Supplement: gkad1158_supplemental_files [file gkad1158_supplemental_files.zip › supplementary_files_legends.docx]

**Supplementary File 1:** List of primers used in this study.

**Supplemental File 2:** Whole transcriptome, differential gene expression analysis comparing miR-1017Δ compared to w^1118^ flies using the Bioconductor package, DESeq2. The first column is gene flybase accession number, followed by DESeq2 calculations (base mean expression, log2 fold change, lfcSE, stat, p-value, and p-adjusted value). The final column indicates (yes or no) whether a transcript is a predicted target of miR-1017.

**Supplemental File 3:** Whole transcriptome, differential gene expression analysis comparing trans-heterozygous miR-1017 mutants and w^1118^ flies using the Bioconductor package, DESeq2. The first column is gene flybase accession number, followed by DESeq2 calculations (base mean expression, log2 fold change, lfcSE, stat, p-value, and p-adjusted value). The final column indicates (yes or no) whether a transcript is a predicted target of miR-1017.
